# *Drosophila* RISC Component VIG and Its Homolog Vig2 Impact Heterochromatin Formation

**DOI:** 10.1371/journal.pone.0006182

**Published:** 2009-07-08

**Authors:** Elena Gracheva, Monica Dus, Sarah C. R. Elgin

**Affiliations:** 1 Department of Biology, Washington University in St Louis, St Louis, Missouri, United States of America; 2 Cold Spring Harbor Laboratory, Cold Spring Harbor, New York, United States of America; University of Munich and Center of Integrated Protein Science, Germany

## Abstract

Heterochromatin formation plays an important role in gene regulation and the maintenance of genome integrity. Here we present results from a study of the *D. melanogaster* gene *vig*, encoding an RNAi complex component and its homolog *vig2* (*CG11844*) that support their involvement in heterochromatin formation and/or maintenance. Protein null mutations *vig^EP812^* and *vig2^PL470^* act as modifiers of Position Effect Variegation (PEV). VIG and Vig2 are present in polytene chromosomes and partially overlap with HP1. Quantitative immunoblots show depletion of HP1 and HP2 (large isoform) in isolated nuclei from the *vig^EP812^* mutant. The *vig2^PL470^* mutant strain demonstrates a decreased level of H3K9me2. Pull-down experiments using antibodies specific to HP1 recovered both VIG and Vig2. The association between HP1 and both VIG and Vig2 proteins depends on an RNA component. The above data and the developmental profiles of the two genes suggest that Vig2 may be involved in heterochromatin targeting and establishment early in development, while VIG may have a role in stabilizing HP1/HP2 chromatin binding during later stages.

## Introduction

The nuclear content of a cell can be roughly divided into two categories: euchromatin and heterochromatin. Euchromatin, where most of the actively transcribed genes reside, is composed of a relaxed array of nucleosomes with corresponding epigenetic marks [1]. Heterochromatin, in contrast, is relatively condensed, as a result of interactions between biochemically modified histone tails and characteristic non-histone proteins, the components of a repressive chromatin assembly. The most prominent heterochromatic mark is histone 3 di- and tri-methylation at lysine 9. This histone modification is found in a large variety of organisms and provides a platform for Heterochromatin Protein 1 (HP1) binding. HP1 recognition of the H3K9 methyl mark and interactions between the H3K9 methyltransferase Su(var)3-9 and HP1 are thought to enable spreading of heterochromatin over significant distances and to explain the existence of vast heterochromatic territories at centromeres. While the interaction of HP1 and H3K9me2/3 appears to be a consistent feature of constitutive heterochromatin, variations on this theme may be found to promote silencing in other contexts (reviewed in [2]–[4]).

The level of chromatin condensation appears to be critical for appropriate regulation of gene expression. When a gene is moved from its normal location to a domain with a different chromatin density, the result is a mosaic gene inactivation, known as Position Effect Variegation (PEV). In *Drosophila* several genes with visible phenotypes, for which inactivation does not affect viability, serve as excellent PEV reporters. Among these, the *white* gene is probably the most heavily exploited. Several genetic screens using a variegating *white* gene as a reporter have identified mutations that enhance or suppress PEV. This has allowed discovery of key structural and regulatory components of heterochromatin, including Su(var)3-9. Additional analysis of PEV modifiers has led to an estimate that as many as 150 genes affect chromatin-related gene silencing [5]. However, so far only a small portion of these genes have been thoroughly investigated.

An unanswered question is how heterochromatin formation is targeted and maintained through cell division. In *S.pombe* heterochromatin assembly at the centromere regions has been linked to transcription of the centromeric repeats. The process of silencing involves interactions between nascent transcripts processed by the RNAi system and the constitutive components of heterochromatin [6]. In this organism, an elegant ‘self-reinforcing loop’ model explains the specificity of heterochromatin formation based on the role of the RNAi machinery (reviewed in [7]–[9]).

RNA interference, discovered less than a decade ago [10], has quickly became recognized as an important regulator of gene expression from plants to worms and humans. Besides the enzymatic core comprised by the protein Ago2, the RNA Induced Silencing Complex (RISC), purified from *Drosophila* S2 cells, contains dFMRP, VIG and Tudor-SN [11]–[13]. Mutations in *ago2* and *dfmr1* have been shown to affect PEV in flies, suggesting a possible role in heterochromatin formation [14], [15]. Suppression of PEV by a gene mutation is an ‘output’ indicating that the silencing chromatin structure is not properly formed. Questions remain as to whether this misregulation occurs at initiation or maintenance, whether the gene product is physically involved in heterochromatin formation, or whether the PEV outcome is a result of indirect effects. The RNAi effector protein Ago2 is associated with endogenous siRNAs targeting some transposons and protein coding genes in germ line and somatic cells [16]–[18]. Another member of Argonaute family, Piwi, is involved in piRNA generation in gonads [19]; *piwi* mutations suppress PEV and Piwi protein can interact with HP1 directly [20], [21]. The large majority of piRNAs (Piwi complexes) and to a much less extent some endo siRNAs (Ago2 complexes) are derived from transposable elements and regulate their silencing in different compartments [16], [17], [22]–[24]. In *Drosophila* up to 77% of the recently annotated 24 Mb of heterochromatic sequences are classified as repetitive transposable elements (TEs). Among these, the retrotransposons (LTRs and LINEs) are the largest group [25] While much of the silencing due to RNAi is accomplished by a post-transcriptional mechanism, it is tempting to speculate that an RNAi-based mechanism may well be an essential part of targeting and maintaining heterochromatic structure in flies.

Among the Ago2- RISC accessory components, VIG belongs to a family of proteins with a recognizable RNA binding motif (the PAI-RBP1 family) that was first identified in a HTC rat hepatoma cell culture. Computational analyses have revealed related proteins in other species including *Homo sapiens*, *Drosophila melanogaster*, and *Arabodopsis taliana*, but not in single cell organisms [26].

The *D. melanogaster* genome contains three genes that encode proteins with the PAI-RBP1 motif: VIG, CG11844 (which we will refer to as Vig2) and CG15031 [26]. *Vig* (*vasa intronic gene*) is located in an intron of *vasa* on the second chromosome and copurifies with Ago2 in a complex containing small RNAs [12]. Mutations disrupting *vig* cause up-regulation of expression from some retrotransposons in *D.melanogaster* ovaries [27], suggesting the possibility of involvement in endogenous siRNA regulation as a member of an Ago2 complex. Defects in the antiviral responses of *vig* mutant flies have also been reported [28]. In contrast very little is known about *vig2* (third chromosome) and *CG15031* (X chromosome), although CG15031 has been reported to act as a protein phosphatase Y-interacting protein [29].

Here we examine the role of VIG and its close homolog Vig2 in heterochromatin formation. We find that while mutations in both genes result in suppression of PEV, it appears that the two proteins impact heterochromatin structure through different mechanisms.

## Results

### Developmental expression profile of *vig* and *vig2*

**Figure 1:**
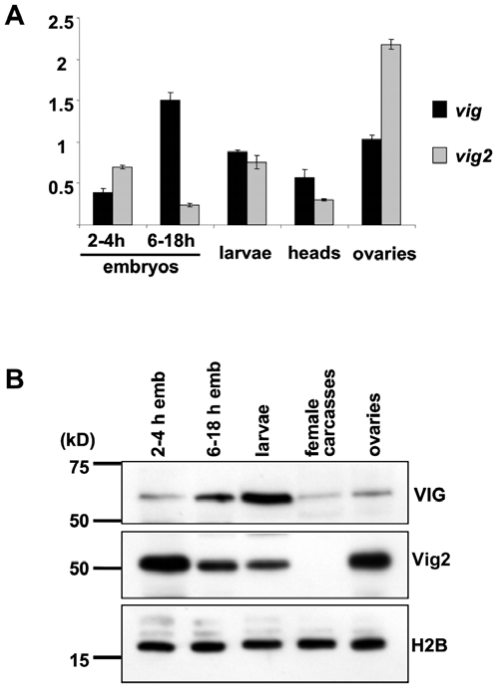
A developmental profile of expression of the *vig* and *vig2* genes generated using quantitative real-time RT PCR and Western immunoblotting. (A) *Vig* and *vig2* are transcriptionally active throughout development reaching the highest levels in ovaries (*vig2*) and in larvae (*vig*). *Vig* and *CG11844* expression levels shown are normalized to the expression of the *RpL32* gene. (B) VIG protein can be detected at all stages of development and in somatic as well as in germline tissue (Western blot using CSH1801). Significant protein accumulation occurs in late embryogenesis and at the larval stage. Vig2 protein is at its highest levels in ovarian tissue and early embryos, but is present throughout development; the amount of protein gradually declines and becomes undetectable in adult soma (Western blot using CSH2542). H2B antibodies were used for the loading control.

VIG and Vig2 proteins are very similar in amino acid sequence (Figure S1), suggesting possible overlapping roles. To study *vig* and *vig2* we generated developmental profiles of RNA and protein levels (Figure 1). Specifically, we were interested in studying material from the developmental stages when heterochromatin formation occurs. Some of the genes involved in RNAi and implicated in heterochromatin formation (*piwi*, *homeless*) are preferentially expressed in the germline and the proteins are maternally loaded. Heterochromatin formation begins at the blastoderm stage at approximately 1.5 hours of embryogenesis [30]. A model proposed by Eissenberg [31] suggests that it is stably maintained during mitotic proliferation from mid-embryogenesis to 3^rd^ instar larvae, and than becomes more relaxed in the post-mitotic period (pupal and adult stages).

We examined *vig* and *vig2* expression in early embryos (2–4 h), late embryos (6–18 h), larvae (2^nd^ and 3^rd^ instars), and adults (somatic tissue and germline) using both real-time RT PCR and Western blots. To detect VIG and Vig2 proteins we generated polyclonal peptide antibodies. Anti-VIG (CSH1801) identifies a specific band of ca 60 kDa on a Western, consistent with the previously reported molecular weight [12]. Anti-Vig2 (CSH2542) recognizes a protein of ca 50 kD. The specificity of the antibodies was verified *in vivo* by gene knock down in *Drosophila* cell culture (Figure S2). We observed differences in the expression patterns of *vig* and *vig2* genes (Figure 1). *Vig2* is activated earlier in development, with transcription peaking in ovaries, and accumulation of the protein in the germline and early embryo. Later the gene is transcribed at lower levels and the amount of protein declines. VIG is also present in the ovaries and early embryos, but transcription peaks at the late embryonic stage; protein accumulation starts in the late embryo and reaches its maximum amount in larvae, when the amount of Vig2 is reduced. While VIG protein is still present in adult somatic tissue, Vig2 is not detected.

### Cellular localization of VIG and Vig2

**Figure 2:**
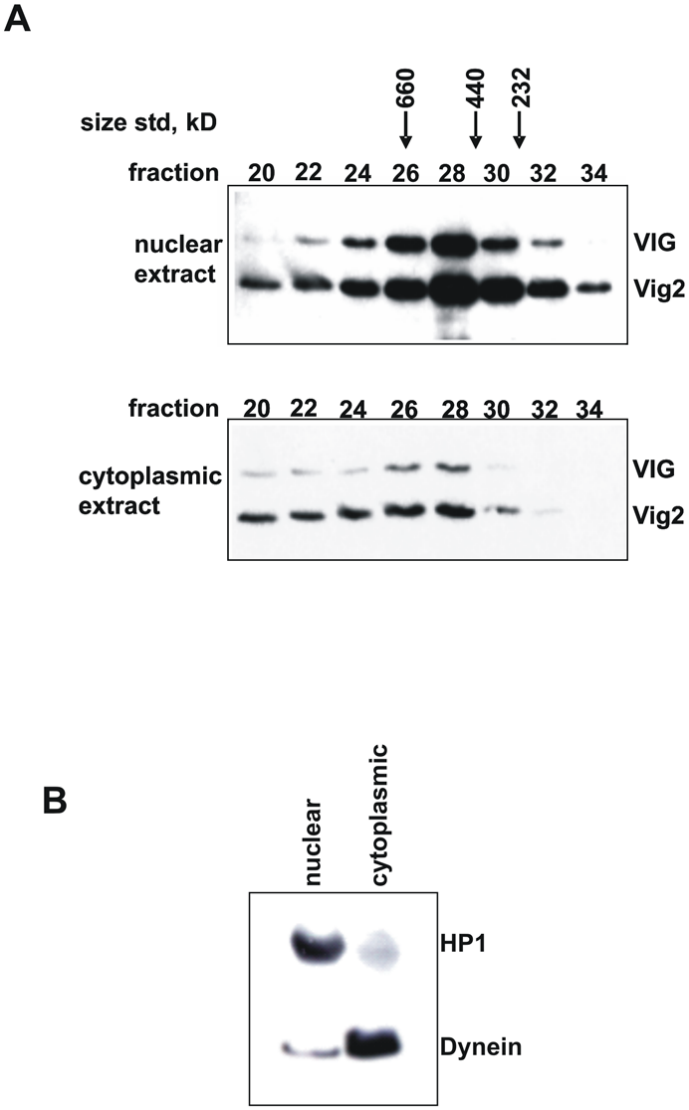
Recovery of VIG and Vig2 from nuclear and cytoplasmic S2 cell extracts. (A) Nuclear and cytoplasmic material was subjected to Superose-6 column chromatography. The numbers above the Western image represent different fractions. Peaks of size standards used to calibrate the column are shown. The Western blot was performed using an antiserum that recognizes both VIG1 and Vig2 (CSH1803). (B) Segregation of nuclear and cytoplasmic components in the extracts used for column chromatography is verified by a control Western blot for HP1 (nuclear) and Dynein (cytoplasmic).

For better functional characterization we performed a partial purification of VIG and Vig2 proteins from nuclear and cytoplasmic material of *Drosophila* S2 cells. We found both proteins in nuclear and in cytoplasmic extracts (Figure 2). These results suggest that VIG and Vig2 function both in the nucleus and in the cytoplasm. The broad peak of VIG and of Vig2 in the nuclear and cytoplasmic fractions suggests that in each compartment these proteins are likely to be associated with other factors in complexes of different sizes and possibly of different functions.

### *Vig* and *vig2* mutations are modifiers of variegation

**Figure 3:**
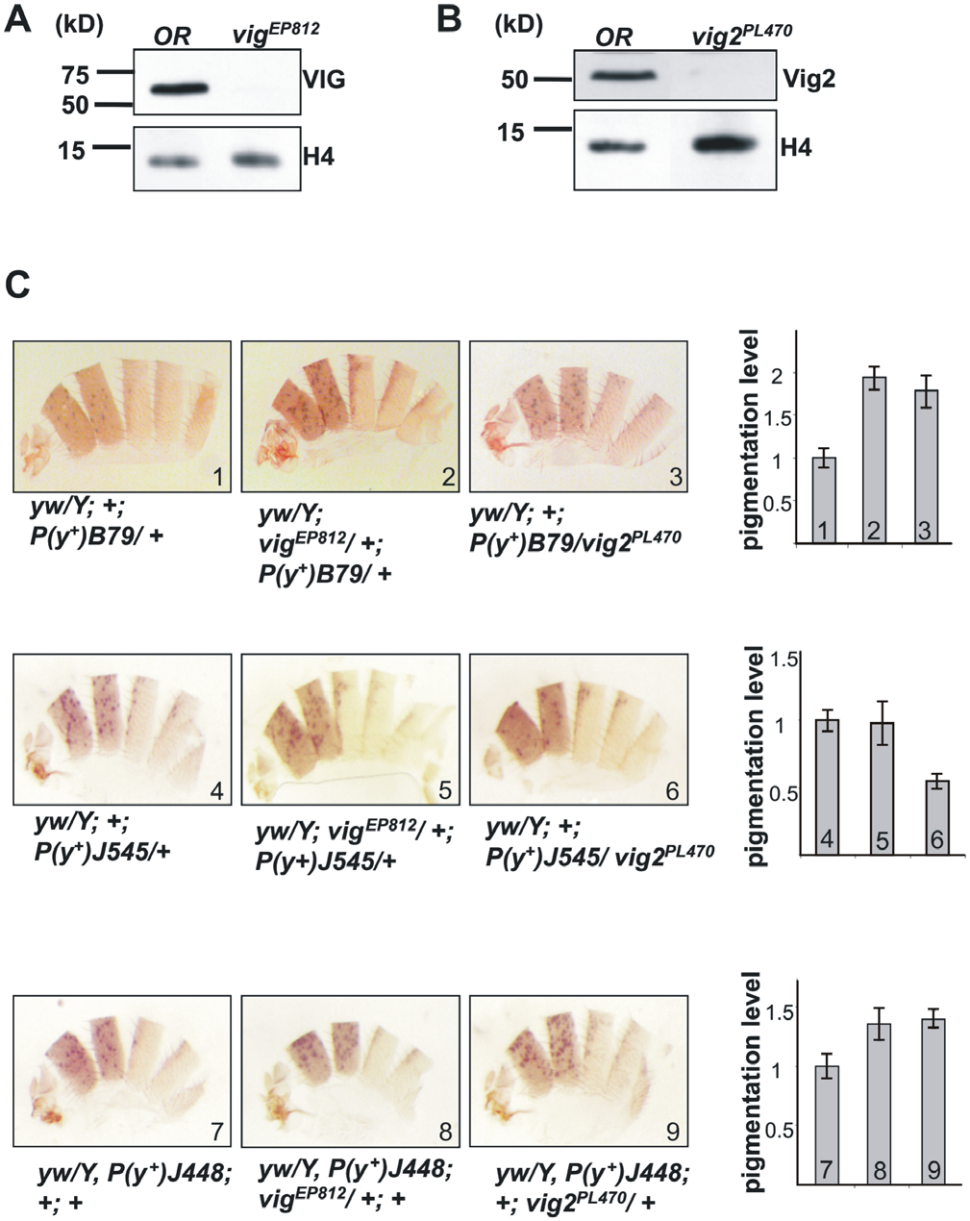
*Vig* and *vig2* mutants are dominant modifiers of PEV. (A, B) VIG and Vig2 production is disrupted in mutant lines as shown by Western analysis. *Vig^EP812^* is a *P*-element insertion in the coding region of *vig*; *vig2^PL470^* is a *piggyBac*-based insertion in *vig2*. Protein extracts from adult flies homozygous for these mutations were used as starting material. (C) Examination of the *yellow* variegating reporters B79, J545, and J448 in a *vig* or *vig2* haplo deficient background. Bar graphs represent the number of pigmented patches on males' dissected abdomens normalized to the control (set at 1). Error bars indicate standard errors. Typical images of the observed phenotypes are shown above the graph.

The PEV system provides a very sensitive assay for the involvement of candidate genes in heterochromatin formation. In this study we used *yellow* variegating reporters located in regions 57h (B79) and 49h (J545) of chromosome 3, and within centric region 10h of chromosome Y (J448) [32], [33] to evaluate the ability of *vig* and *vig2* mutations to affect PEV. The *vig^EP812^* mutation is caused by a P{EP} insertion within the *vig* coding region (Figure S3A) that results in severe reduction of the VIG protein (Figure 3A). PBac{GAL4D,EYFP} resides in the *vig2* gene [34] and disrupts the production of Vig2 protein (Figure 3B). We generated flies carrying the *yellow* PEV reporters and mutations in either *vig* or *vig2*:

*y^1^w^67c23^/Y; vig^EP812^/+;* ***P(y^+^)B79*** */+*

*y^1^w^67c23^/Y; +;* ***P(y^+^)B79*** */vig2^PL470^*

*y^1^w^67c23^/Y; vig^EP812^/+;* ***P(y^+^)J545*** */+*

*y^1^w^67c23^/Y; +;* ***P(y^+^)J545*** */vig2^PL470^*

*y^1^w^67c23^/Y,* ***P(y+)J448*** *; vig^EP812^/+; +*

*y^1^w^67c23^/Y,* ***P(y+)J448*** *; +; vig2^PL470^/+.*

To score the variegating *yellow* phenotype we prepared male abdominal cuticles and compared the number of dark patches to the WT control. The control flies were obtained simultaneously in a separate cross between males carrying the reporter and *y^1^w^67c23^* females (for example: *y^1^w^67c23^/Y*; *+*; *P(y^+^)B79/+*). (This control cross is necessary because it is inappropriate to use siblings carrying the PEV reporter and a balancer chromosome as a WT control, as the balancer chromosomes often carry modifiers of PEV). However, the *vig^EP812^* mutation affects the *vasa* gene as well as *vig* (Figure S3A), and any suppression of PEV in this case could be attributed to the deficit in Vasa protein. To resolve this issue we crossed a stock carrying the *yellow* PEV reporter *P(y^+^)B79/+* and the *vasa^AS^* mutant line (Figure S3A), in which only the *vasa* gene is affected [35] and *vig* expression is at wild type level (Figure S3B). Analysis of the progeny phenotype demonstrated that the *vasa^AS^* mutation does not modify PEV (Figure S3C), indicating that the suppression observed with the *vig^EP812^* mutation is caused by a reduction in the levels of VIG.

Silencing of the *yellow* reporter B79 was relieved in a *vig^EP812^* or *vig2^PL470^* heterozygote mutant background (Figure 3C, panels 1–3). The J545, reporter located in a different heterochromatic domain, was not sensitive to the *vig^EP812^* mutation; but *vig2^PL470^* resulted in enhancement of variegation (Figure 3C, panels 4–6). The J448 P-element is inserted in the centric region of the Y chromosome; flanking sequences include *1360* and *Su(Ste)*. Both *vig^EP812^* or *vig2^PL470^* mutations caused suppression of variegation at this location (Figure 3C, panels 7–9). The differences seen with various PEV reporters are not without precedent; *Su(var)3-9* mutations result in suppression of variegation for reporters in the pericentric heterochromatin, but enhancement of variegation for reporters in the fourth chromosome [36], [37]. Overall, the results of the *yellow* PEV tests suggest an involvement of VIG and Vig2 proteins in heterochromatin formation or maintenance. However, the PEV reporters' readouts indicate that VIG and Vig2, unlike HP1, are not heterochromatin ‘construction material’; our data suggest that *vig2* gene is rather involved in targeting (nucleation) of heterochromatic domains. Mutations in this gene may cause the disruption of proper distribution of heterochromatin structural components; as a result we observe suppression or enhancement of variegation. One can speculate that Vig2 is epistatic to VIG in this process. Vig2 is prominent in the early embryo, when heterochromatin formation is initiated, while VIG is expressed at higher levels in larval stages, when heterochomatin formation must be maintained.

### VIG and Vig2 partially overlap with HP1 on polytene chromosomes

Large polytene chromosomes are found in the larval salivary glands of *Drosophila*. Immunostaining of these chromosomes with antibodies allows detection and analysis of the distribution pattern of any nuclear protein *in vivo*. HP1 stains predominantly the chromocenter, telomeres, and the small 4^th^ chromosome, the domains where most of the heterochromatin is found. In addition, HP1 also marks numerous sites on the chromosome arms, including the ‘goose neck’, a cluster of bands on the 2^nd^ chromosome [38], and large puffs (sites of intense transcription) [39].

**Figure 4:**
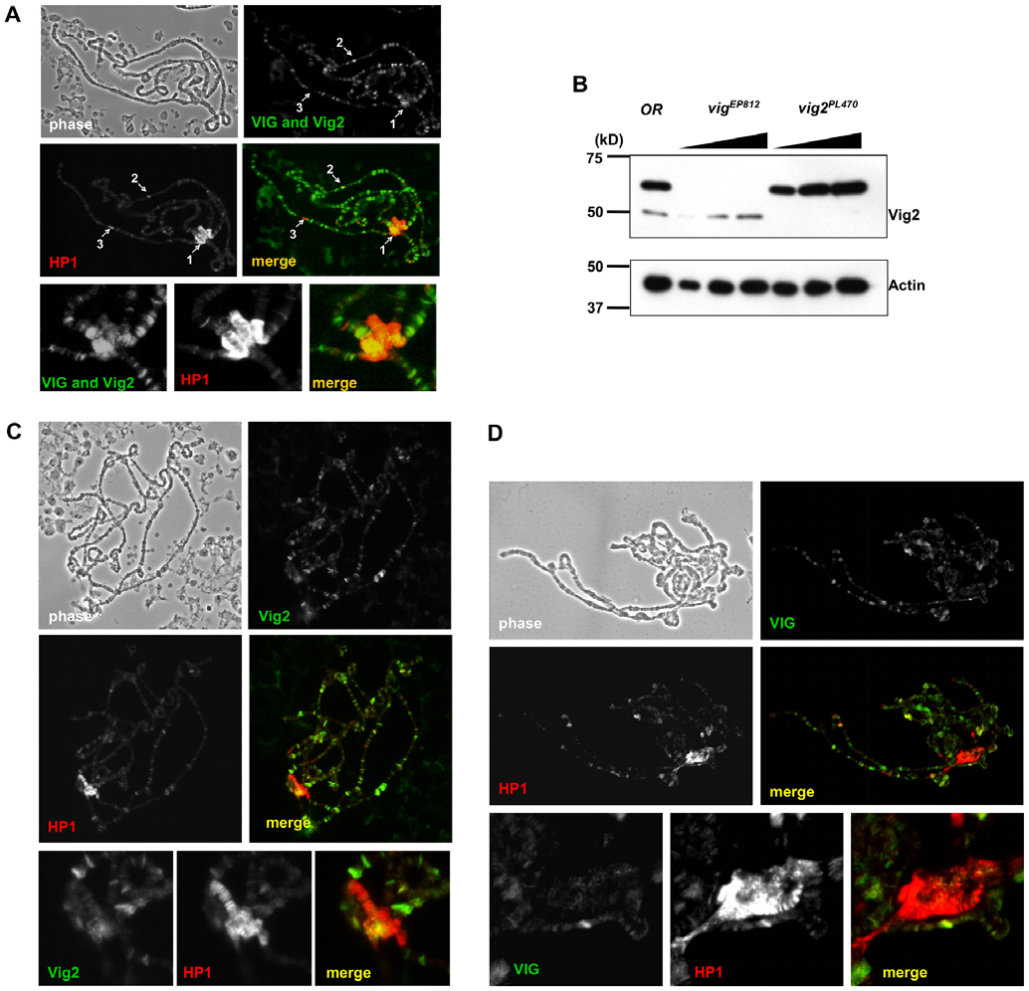
Immunofluorescent staining of polytene chromosomes. (A) VIG and Vig2 (visualized using CSH1803) show a distribution pattern that overlaps with HP1 at the chromocenter *(arrow 1)* and in some cases on the chromosome arms *(arrow 2)* of the WT polytene chromosomes. However, the majority of the cytobands do not overlap; for instance, region 31 on the 2nd chromosome (the ‘goose neck’) is associated with HP1 alone *(arrow 3)*. The lower row of images shows staining at the chromocenter. (B) Western blot performed using protein from salivary glands shows detection of both VIG and Vig2 protein by the CSH1803 antibody. In the *vig^EP812^* mutant only Vig2 is present; in the *cg11844^PL470^* mutant only VIG is recognized. (C) Polytene chromosomes were obtained from *vig^EP812^* mutant larvae; in the absence of VIG we observe significant overlap between HP1 and Vig2 protein at the chromocenter and at some sites on the chromosome arms. The lower row of images shows staining at the chromocenter. (D) The distribution of VIG seen on *vig2^PL470^* mutant polytene chromosomes shows multiple overlapping sites shared by HP1 and VIG on the chromosomal arms, but only faint speckles of VIG at the chromocenter. The lower row of images shows staining at the chromocenter.

To visualize the distribution of VIG and Vig2 relatively to HP1 we treated WT polytene chromosomes with antibodies generated against the C-terminus of VIG (CHS1803) and a mouse monoclonal antibody specific for HP1 (C1A9) (Figure 4A). CHS1803 recognizes both VIG and Vig2; it was used in preference to other VIG antibodies because of its high sensitivity and the absence of cross-reacting bands on a Western blot using total protein from salivary glands (Figure S4). The CSH1803 antiserum stains the chromocenter and produces a complex pattern on the chromosome arms. Figure 4A shows the overlap between HP1 signal and the signal produced by CSH1803 at the chromocenter (*arrow 1*) and at some sites on chromosome arms. Only partial overlap is observed; for example, the ‘goose neck’ region, prominently associated with HP1, is devoid of both VIG and Vig2 (*arrow 3*). Among numerous sites on the chromosomal arms marked by CSH1803, only some are shared with HP1 (*arrow 2*). To obtain the individual distribution patterns of VIG and Vig2, we used salivary glands from homozygous *vig^EP812^* or *vig2^PL470^* mutant larvae, after having verified by immunoblot (Figure 4B) that in the absence of VIG only Vig2 is recognized by this antibody and vice versa. The distribution pattern of Vig2 seen on *vig^EP812^* polytene chromosomes (Figure 4C) clearly shows overlap between HP1 and Vig2 at the chromocenter and at some sites on the chromosome arms, including sites of active transcription (large puffs). By contrast, VIG staining of *vig2^PL470^* chromosomes presents different features (Figure 4D). There is little signal present at the chromocenter and intense staining is seen at scattered sites on the chromosome arms, including some large puffs where HP1 is also present. Thus, VIG localization on polytene chromosomes differs from that of Vig2. In particular, Vig2 association with constitutive heterochromatin domains suggests that this protein could be involved in heterochromatin formation and/or maintenance. On the other hand, VIG overlaps with HP1 primarily at some sites on the chromosome arms, including puffs, suggesting either a direct role in heterochromatin formation or participation in regulatory processes that have indirect effects on heterochromatin.

### The H3K9me2 level is decreased in the *vig2^PL470^* mutant, but not in *vig^EP812^*

**Figure 5:**
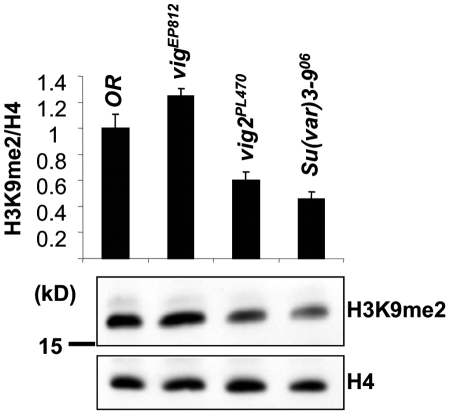
Quantitative Western blot for H3 dimethylation at Lys9. Total protein extracts were obtained from adult flies and probed with an anti-H3K9me2 specific antibody; anti-H4 was used as a loading control. A stock carrying a null mutation of *Su(var)3-9* histone methyltransferase was used as a control. *Vig2^PL470^* demonstrates a diminished level of H3K9me2, whereas *vig^EP812^* does not. Error bars represent standard error of the mean.

To further investigate the role that VIG and Vig2 play in the process of heterochromatin formation, we looked at the hallmark of silent chromatin - H3K9 dimethylation. We prepared protein extracts from adult flies and assessed the level of H3K9me2 by quantitative Western blot (Figure 5). For reference we used the H3K9me2 levels of the *Oregon R* (WT) strain and the *Su(var)3-9^06^* mutant flies. The *Su(var)3-9* gene encodes one of three known histone H3K9 methyltransefases in *Drosophila*, and is an effective suppressor of PEV. *Su(var)3-9^06^* is a null allele in which H3K9me2 levels are significantly decreased in embryos [40]. In adult flies the dimethylation of H3K9 in a homozygous *Su(var)3-9* mutant background fell by ca. 50% relatively to WT. The homozygous *vig^EP812^* mutant did not show any loss of H3K9 methylation, but the homozygous *vig2^PL470^* mutant clearly demonstrated a decrease to ca. 60% of WT levels. We also checked different types of tissues. Adult heads (soma) and ovaries (germline) both exhibit the same pattern of H3K9me2 depletion as seen in whole adult flies (Figure S5). In conclusion, while both *vig* and *vig2* mutants can be dominant suppressors of PEV, these experiments show that *vig2* impacts H3K9 methylation, a key signature of heterochromatin, while *vig* does not.

### HP1 and HP2 (Large isoform) are depleted in the nuclei of a *vig* mutant

**Figure 6:**
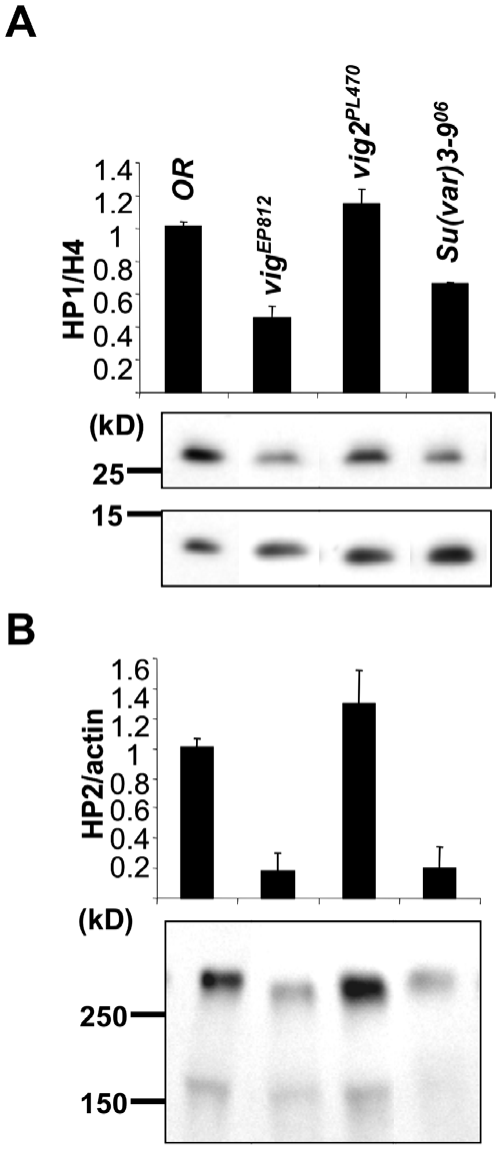
Quantitative Western blots to estimate the amounts of HP1 and HP2 in adult fly nuclear preparations. (A) The *vig^EP812^* strain shows a decrease in HP1 similar to the *Su(var)3-9^06^* mutant. In *vig2^PL470^* flies the level of HP1 appears to be similar to WT. H4 was used as a loading control. (B) The HP2 Large isoform follows the HP1 pattern. Actin served as a loading control. Error bars represent standard error of the mean.

Next we extended our analysis to another major component of heterochromatin, the HP1 protein. Nuclear proteins were extracted from adult tissues of the same fly strains: *Oregon R* (WT), *vig^EP812^*, *vig2^PL470^*, and *Su(var)3-9^06^*. Because prior studies have shown that in homozygous *Su(var)3-9^06^* mutant larvae HP1 is lost from the chromocenter of polytene chromosomes, we used this mutant line as a positive control [41]. As expected, quantitative Western blots show a loss of nuclear HP1 in *Su(var)3-9^06^* mutants. Similarly, we detected only about 50% of the WT level of HP1 in the homozygous *vig^EP812^* mutant line, but we did not see any changes in homozygous *vig2^PL470^* flies (Figure 6A). We considered the possibility that the *vig* mutation could affect the expression of *Su(var)205* (most likely indirectly) and tested *Su(var)205* RNA levels by RT-PCR. However, there were no changes in a comparison to the levels of *Su(var)205* messenger RNA in WT and *vig^EP812^* mutants (Figure S6). To further verify the HP1 results, we looked at a binding partner of HP1, Heterochromatin Protein 2 (HP2) [42], [43]. The *Su(var)2-HP2* gene codes for two HP2 protein isoforms, HP2 Large (HP2L) and HP2 Small (HP2S); HP2L binding to heterochromatin is HP1-dependent. We estimated the amount of HP2L in nuclei by quantitative Western blot (Figure 6B). The results obtained for HP2L were reflective of HP1: loss of HP2L was observed in nuclei prepared from *vig^EP812^* and *Su(var)3-9^06^* mutant flies, but not in those from the *vig2^PL470^* line. These results suggest that VIG might be involved in stabilizing the interaction of HP1 with chromatin, while Vig2 does not play such a role. The latter result is surprising, given that the Vig2 mutation has an effect on H3K9 methylation.

### HP1, VIG and Vig2 co-immunoprecipitate

**Figure 7:**
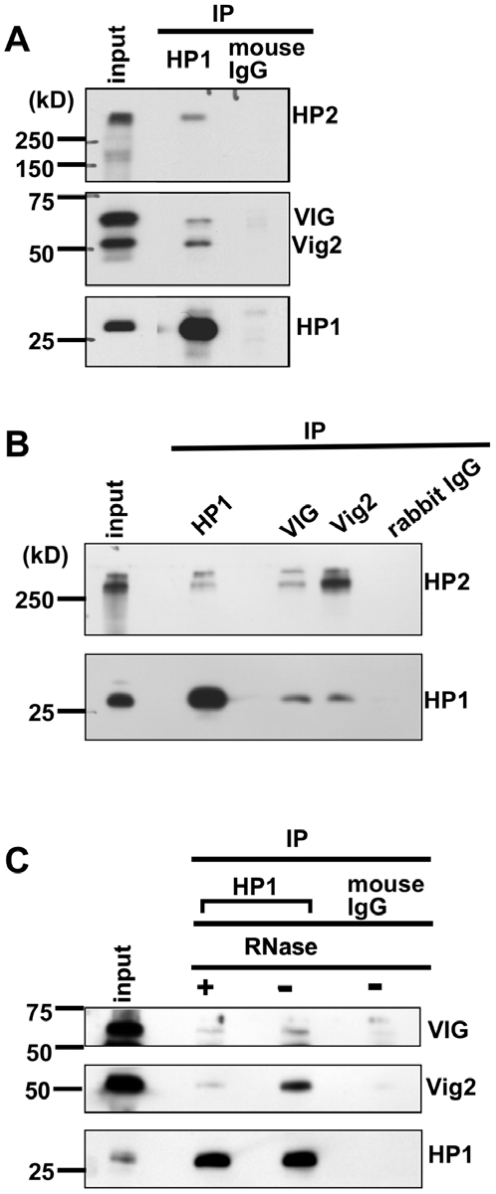
Co-immunoprecipitation experiments show the association of heterochromatin structural proteins HP1 and HP2 with the RNA-interacting proteins VIG and Vig2. (A) The HP1 monoclonal antibody C1A9 pulls down HP2 large isoform (as expected) and both VIG and Vig2 proteins. Mouse IgG was used for the control mock immunoprecipitation. (B) Antibodies specific for VIG (CSH1801) and Vig2 (CSH2542) proteins pull down HP2 Large isoform and HP1. Rabbit IgG was used as a negative control. (C) RNaseA treatment of the homogenates reduces the amount of VIG and Vig2 protein pulled down by the HP1 antibody. Mouse IgG was used for the control mock immunoprecipitation.

To examine whether VIG or Vig2 are present in a complex with HP1 we performed immunoprecipitation experiments using antibodies specific for HP1 (C1A9 and WA191), VIG (CSH1801), and Vig2 (CSH2542). In addition to co-precipitating HP2L, as expected, HP1 also co-precipitated VIG and Vig2 (Figure 7A). Similarly anti-VIG and anti-Vig2 antibodies pulled down both HP1 and HP2L (Figure 7B). Because VIG and Vig2 contain RNA binding domains, we tested whether these interactions with HP1 were dependent on an RNA component. Indeed, treatment with RNase A prior to immunoprecipitation with anti-HP1 antibodies resulted in loss of signal, suggesting that the HP1/ VIG and HP1/Vig2 interactions are RNA dependent.

## Discussion

We have presented new results suggesting the involvement of the RNAi-associated protein VIG, and its homolog Vig2, in heterochromatin formation. VIG and Vig2 belong to the PAI-RBP1 family. Based on DNA sequence and protein database analyses, one can infer that these proteins play a role in mRNA stability and/or in processes requiring interactions with RNA [26]. Human paralogs of VIG, SERBP1 and HABP4, have been reported to localize in the cell cytoplasm, nucleus, and perinuclear regions; our data indicate that VIG and Vig2 are both nuclear and cytoplasmic (Figure 2). Human VIG paralogs interact with the silencing chromatin remodeling complex CHD3, as shown by a yeast two-hybrid screen [44]. Our data expand the proposed role of PAI domain proteins in chromatin regulation by demonstrating that deficits in VIG and Vig2 can result in suppression of PEV (Figure 3). We observe both *in vivo* co-localization (Figure 4) and evidence of interaction (Figure 7) with major components of repressive chromatin, HP1 and HP2.

It has recently been reported that in *Drosophila*, HP1 binding and H3K9 methylation in heterochromatin are dependent on physical interaction with ‘inactive’ (unphosphorylated) STAT [45]. This is a surprising connection, since canonical JAK/STAT regulation pathways have been thought of as generally involved in transcription regulation. VIGs' human paralog SERBP1 interacts with Protein Inhibitor of Activated STAT (PIAS) [46], which has its own fly paralog encoded by the *Su(var)2-10* gene [47]. These pieces of information suggest an interpretation of our results. Our data demonstrate that VIG is involved in stabilization of HP1 binding to chromatin (Figure 6) and that Vig2 affects H3K9me2 levels (Figure 5). Considering the findings discussed above, one can speculate that in *Drosophila* VIG and Vig2 might function in concert with components of the JAK/STAT pathway or a non-canonical STAT pathway to modulate heterochromatin formation. In particular, RNA binding proteins such as VIG, perhaps collaborating with Su(var)2-10, a PIAS protein, could potentially facilitate binding of inactive STAT and consequently HP1 to heterochromatic domains. The *Drosophila* JAK/STAT pathway has an important role in the immune response, particularly in viral defense [48], a process also related to RNAi. Our results indicate that the association between VIG or Vig2 and HP1 is RNA dependent. One can speculate that transcripts originating from transposable elements (a major sequence component of heterochromatin), processed by the RNAi machinery, could be the constituents of a targeting complex containing HP1. VIG has been demonstrated to interact with small RNAs in *Drosophila* S2 cells. The human paralog of VIG has been shown to interact with L1 retrotransposon products in the stress granules and to participate in regulation of retrotransposition together with other RNAi components, including Ago2 and FMRP [49]. However, *Drosophila* VIG and Vig2 -interacting RNAs have yet to be identified and characterized.

Many unanswered questions still remain. Why do deficits of either VIG or Vig2 have such different impacts on HP1 nuclear binding and H3K9 methylation? Many previous studies have shown that HP1 binding and H3K9 methylation are interdependent [40]. However, detailed analyses of HP1 distribution using cytoimmunochemical approaches and high resolution mapping based on the DamID technique have shown that although HP1 and the H3K9me2 mark generally overlap in the chromocenter and pericentric regions, there are also numerous sites on the chromosome arms where HP1 binding is not associated with di- or trimethylation of H3K9. In some cases this binding is dependent on RNA [39], [50], [51]. Our cytological results indicate that VIG and HP1 overlap mostly outside of the chromocenter, including sites of active transcription (Figure 4D). Destabilization of HP1 binding in those locations seems unlikely to result in significant overall changes in H3K9me levels. The HP1/VIG immunosignals could mark sites of endogenous small RNA precursors and could be involved in regulatory processes affecting heterochromatin indirectly.

In the *Su(var)205* mutant, the resulting HP1 deficit does not result in loss of H3K9 methylation; Su(var)3-9 HKMTase and H3K9me2 are still present at the chromocenter, but are also found in euchromatin, resulting in an almost ubiquitous distribution of H3K9me2 [41]. This suggests that HP1 is probably not the only protein involved in tethering HKMTases to chromatin, although a role for HP1 in sequestering H3K9 methylation to heterochromatin is clear [52]. Therefore our results showing that *vig* mutation causes HP1 destabilization without an effect on H3K9 methylation level is not without precedent.

The developmental profile generated for *vig* and *vig2* expression shows that the two genes have activity peaks at different stages of development. Vig2 is very abundant in ovaries, where several different HKMTases (dSETDB1, Su(var)3-9, and G9a) work together to methylate H3K9 [53]–[55]. Depletion of Vig2 results in a decreased level of H3K9me2 in adult flies; however, there was no effect on the amount of HP1 observed in the nuclei. Of the three HKMTases, only Su(var)3-9 is known to recruit HP1 molecules directly, while the others utilize alternative mechanisms, yet to be identified. The point of intersection with Vig2 remains to be identified.

Despite the differences in their action, both VIG and Vig2 appear to be new components of an extensive network embracing heterochromatin, small RNAs and transcription regulation. Studying the specific functions of VIG and Vig2 in detail will be important in understanding the dynamics of heterochromatin establishment and maintenance.

## Materials and Methods

### Fly strains and genetic experiments

Fly stocks were maintained at 22°C, 70% humidity on a cornmeal sucrose-based medium [56]. The w*;P{EP}vig^EP812^/CyO stock was obtained from the Szeged Drosophila Stock Centre; homozygous mutant flies are viable, females are sterile. w^*^; P{FRT(w^hs^)}2A P{neoFRT}82B PBac{GAL4D,EYFP}CG11844^PL00470^ (*vig2^PL470^*) was obtained from the Bloomington Drosophila Stock Center (BL #19518); homozygous mutant flies are viable and fertile. *vas^AS^* is described in [35]. X chromosomes of the above mentioned lines were replaced by the *y w* chromosomes. In the *vig^EP812^/CyO* stock the 2^nd^ chromosome balancer was changed to *CyO, P{w^+mC^ GAL4}, P{w^+mC^ UAS-GFP}* for polytene chromosome immunofluorescence experiments. *w; Su(var)3-9^06^* has been described elsewhere [57]. The PEV reporters B79, J545, J448 have been characterized previously [32], [33].

To assess modification of PEV, genetic crosses were conducted as follows: virgin females carrying the mutation of interest (*y^1^w^67c23^*; *vig^EP812^/CyO*, or *y^1^w^67c23^*; *vig2^PL470^*, or *y^1^w^67c23^*; *vas^AS^/CyO*) or ‘wild type’ *y^1^w^67c23^* flies were crossed to males carrying the PEV reporters *y^1^/Y; +; P(y^+^)B79, y^1^/Y; +; P(y+)J545, y^1^/Y, P(y+)J448; +; +.* Male progeny from WT and mutant mothers were compared.

### Adult abdominal cuticle preparation

Flies were immobilized on the adhesive side of a piece of tape and covered with PBST. The abdomens were sliced off, dissected along the dorsal midline, and cleaned. These abdomen cuticle halves were transferred to a drop of mounting media [Shandon Immu-Mount (Thermo Fisher Scientific) supplemented with chloral hydrate (53% v/v) and lactic acid (9% v/v)], spread on a microscope slide, covered by a cover slip, left to dry overnight on the slide warmer at 42°C, and subsequently photographed.

### Real time RT-PCR

RNA was extracted using TRIzol Reagent (Invitrogen) according to the manufacturer's recommendations. For quantitative real time one-step RT-PCR we used the QuantiTect RT-PCR Kit (Qiagen), following the instructions provided by the manufacturer for the SmartCycler PCR machine (Cepheid). Standard curves were generated using 0.5–500 ng of total RNA for each primer pair. The expression level of the *RpL32* gene was used for normalization.

Primers used: *Su(var)205* (*HP1*): ACCATTTCTGCTTGGTCCAC and CAAGCGAAAGTCCGAAGAAC; *vig*: TTCGCTGTCGTTCTCCTTCTCCTTCT and AAAGAGCTGACCTTGGACGA; *vig2*: GCGTCAATTCAACAATCGTG and CCCGGTCGTCTTTAAGTCCT; *RpL32*: ATGGTGCTGCTATCCCAATC and GTCGCCTGCGTTCTCAAG.

### Antibodies

Rabbit polyclonal antisera directed against the N-terminal 10–14 AA of VIG and Vig2, CSH1801, CHS1803, and CSH2542 were raised as previously described [11]. Anti- HP1 [38] anti-HP1 (WA191 [58]), anti-Vasa (Lasko and Ashburner, 1990) and anti-HP2 [59] have been previously described. Anti-H2B (# 07–371); anti-H3K9me2 (# 07–441); anti-H4 (# 07–108) were purchased from Upstate; anti-Actin (JLA20) was obtained from DSHB; anti-Dynein was purchased from Sigma (D5167).

Secondary HRP-conjugated antibodies were purchased from Thermo Scientific.

### Polytene chromosomes squashes and immunostaining

Polytene chromosomes were prepared as described [60]. VIG deficient 3^rd^ instar larvae were selected based on the absence of fluorescence from *vig^EP812^/ CyO, P{w^+mC^ GAL4}, P{w^+mC^ UAS-GFP}* stock. HP1 distribution patterns were identified using the C1A9 monoclonal antibody diluted 1∶10. VIG and Vig2 were detected using the CSH1803 antibody at a 1∶5 dilution. Secondary antibodies (Molecular Probes) were labeled with Alexa Fluor 594 (red) and Alexa Fluor 488 (green). The images of HP1 and VIG or Vig2 distribution were captured simultaneously (Figure 4, colored panels); then red and green color channels were separated using Corel PHOTO-PAINT software to create the black and white images of HP1, VIG, and Vig2 individual distributions.

### Western analysis

Total protein extracts were obtained as follows: 3–4 day old flies were collected, ground in 10 mM Tris pH 8 with ‘Complete’ Protease Inhibitor Cocktail (Roche), and left on ice for 30′. 2× Laemmli loading buffer (LLB) (BioRad) was added and the mixture boiled for 5 min. Nuclei were prepared as described previously [58]. Nuclear material was sheared using a syringe (22–24G needle) and the homogenate boiled in LLB for 5 min. At least 3 independent protein extractions were performed for each experiment. Extracted proteins were size separated by SDS-PAGE, transferred to a nitrocellulose membrane (Protran, Whatman) and probed with the antibody of interest in 2.5% milk TBS-T overnight at 4°C. The antibodies were diluted as follows: anti-VIG (CSH1801) 1∶5,000; anti-Vig2 (CSH2542) 1∶20,000; anti-VIG (CSH1803) 1∶10,000; anti-H2B 1∶10,000; anti-H3K9me2 1∶20,000; anti-H4 1∶2,000; anti-HP1 1∶100; anti-HP1 (WA191) 1∶ 2,000; anti-HP2 1∶10,000; anti-Actin 1∶500; anti-Vasa 1∶5,000; anti-Dynein 1∶2,000. Secondary goat anti-rabbit (or mouse) HRP-conjugated antibodies were diluted as 1∶100,000–500,000. SuperSignal West Femto Maximum Sensitivity Substrate (Thermo Scientific) was used for detection. Quantitative data was obtained as follows: gradually increasing amounts of the protein extract were loaded in successive lanes for all genotypes and probed with the corresponding antibodies as described above. Densitometry measurements of this dilution series showed that the antibodies applied were sensitive to the dosage of the proteins and that the optical signal response was linear in this range. ‘Standard curves’ showing the optical value dependence on the amount of loaded protein for the WT sample were generated for the protein of interest and for the loading control protein. Using these standard curves we determined the relative amount of proteins in the mutant lines. The bar graphs represent the ratio between the protein of interest and the loading control. (Note that because the two antibodies (one specific for the protein of interest and the other for the loading control) demonstrate different sensitivities and different response curves, it is not appropriate to use un-calibrated optical data for quantitation.)

### Purification of VIG and Vig2 from nuclear and cytoplasmic S2 cell extracts

S2 cells were harvested, washed twice in PBS, resuspended in buffer A (10 mM Hepes pH 7.9, 1.5 mM MgCl2, 10 mMKCl, 1 mM DTT), and protease inhibitors (Roche) and lysed in ice for 30′. Cells were then disrupted in a Dounce homogenizer with the B pestle for 40 strokes and spun at 800 g for 20′. The supernatant was collected and clarified at 20,000 g for 30′; this is the cytoplasmic fraction. The pellet from the 800 g spin was resuspended in a small amount of buffer A plus 2 mM CaCl_2_ and treated with Turbo DNAseI (Ambion) for 2′ in a 37°C water bath with shaking. The nuclei were then spun down briefly and resuspended in buffer B (20 mM Hepes pH 7.9, 40 mM NaCl, 1.5 mM MgCl2, 20 v/v glycerol, 0.2 mM EDTA), and agitated for 30′ at 4°C. The nuclear extract was then homogenized and clarified by spinning at 20,000 g for 30′. The supernatant is the nuclear fraction. The pellet was then sonicated in SDS loading buffer to obtain a chromatin fraction. Both cytoplasmic and nuclear extracts were filtered through 0.4 micron and 0.8 micron filters, respectively.

AKTA FPLC was used for Superose- 6 column chromatography [12], [61]. Two mls of nuclear and cytoplasmic extracts (20–35 mg/ml) were filtered through a 0.4/0.8 micron filter, loaded onto a Sepharose 6 column (Amersham Biosciences) and eluted with 1.2 column volumes of buffer A′ (100 mM NaCl instead of 10 mM KCl). 10 ul from each fraction were boiled with SDS sample buffer for 5 minutes and loaded onto a 10% SDS-PAGE.

### Immunoprecipitation experiments

Whole flies or ovaries were ground in Lysis Buffer (20 mM Tris pH 7.5, 100 mM NaCl, 0.5 mM EDTA pH 8, 0.5% NP40, ‘Complete’ Protease Inhibitor Cocktail (Roche)). Fly homogenates were filtered through 1 layer of Miracloth (Calbiochem). Preclearing step: 50 ul of Protein A/G beads (Thermo Scientific) previously washed in Lysis Buffer were added to 500 ul of fly homogentate and the mixture was incubated on a rocking platform for 30 min at 4°C; the beads were collected by centrifugation (2 min at 500 g) and the liquid phase was recovered. The aliquot taken at this step served as the ‘input’ sample. 5 ug of the antibodies or purified IgG (Jackson ImmunoResearch) was added to the homogenates and the mixtures were incubated on a rocking platform for 2 h at 4°C; then 50 ul of Protein A/G beads were added and incubation continued for 1 h at 4°C. The beads were spun down and washed 3 times with Lysis Buffer. The antibodies and immunoprecipitated material were released from the beads by boiling in 50 ul of LLB for 5 min; the proteins were analyzed by Western blot.

RNase treatment of extracts was conducted as outlined [62]. RNase A (100 ug/ml final concentration) was added to the precleared extracts before immunoprecipitation and the rest of procedure was performed as described above.

## Supporting Information

Figure S1 (0.06 MB PDF)

Figure S2 (0.13 MB PDF)

Figure S3 (0.05 MB PDF)

Figure S4 (0.38 MB PDF)

Figure S5 (0.06 MB PDF)

Figure S6 (0.04 MB PDF)
