# Supplementary material for: Drosophila RISC Component VIG and Its Homolog Vig2 Impact Heterochromatin Formation
Source: PLoS One. 2009 Jul 8;4(7):e6182. doi: 10.1371/journal.pone.0006182 (PMC2703606; doi:10.1371/journal.pone.0006182)
Supplement: Figure S1 — (0.06 MB PDF) [file pone.0006182.s001.pdf]

```
VIG             MDSAGKNRYELLFMDDDVSDPLDNLVAPTAAAAAAAAGKKKQPSAAAAAATKTTANKVAN 60
Vig2            MQLAEKHIVALMFPAIDVSTFTFVSGVKFYIMDNSGNNRYELLFMDDDDSSGLAQPQIAA 60
                *: * *:   *:*   ***       .       :. .: :        ::  :  ::*

VIG             SNNKANAGSNIGGPNAKKPNQAEKENKPNNALNKTDGKKFTPSADNKQQQFNNNASSNYK 120
Vig2            VVAAPKKPEPAKAPKAPK-SKSEKENKPVVAARKAN----APVAKN-------------- 101
                    .:  .   .*:* * .::******  * .*::    :* *.*

VIG             QQGAPRQGGGANRTREFGSGQGQGQGQGGQQQRSVNFRQQNGNAETREQRNNRRNVRENV 180
Vig2            --ASPVKGGKGPAGGDVGRPKNP-TANGANNQGRFNNNQRYGNKESNGEFGNELPQRQ-- 156
                  .:* :** .    :.*  :.   .:*.::*  .* .*: ** *:. : .*.   *:

VIG             GAPDGQQSRPYRGPGGGPGAGGDRPQRQNRNYDGQNRKREFDRQSGSDRTGVKSIDKRDG 240
Vig2            -----FNNRDNRGP-----------PRVRTGEKFG---KREFDRQSGSDRTGVKSIDKREG 198
                      :.*  ***          *: :. :  *   *********************:*

VIG             AGSHNWGSVKEAIDDVNKNESETNVTNAEGGAKADESGTEPQNEQATAEEEAKELTLDEW 300
Vig2            GGAHNWGSPKQDIEDLKTTGETS--PQAEKEDSANEQSADP--AVAAEEDESKQMTLDEW 254
                .*:***** *: *:*::.. . :  .:**   .*:*..::*    *: *:*:*::*****

VIG             KAQQGQRIKPTFNIRKAGEGE-DTTQWKKMVVLTSNKKKENDSEEELEYDPALYPQRVGR 359
Vig2            KALRDQRAKPNYNLRKAGEGAADNAEWKKMIVLS--KKKESNSEDELEYDPSLYPQRVGR 312
                ** :.** **.:*:******  *.::****:**:  ****.:**:******:********

VIG             QQRVLDIQFNFNDGRRGGPGGFGGRGGRGGPRPGGFGG-GPRSEGGNRDGGNR-EGGRDN 417
Vig2            LQRIVDIQFNFNDGRKVGFR-KGPRPG-AGPREGGFRNDGPRGEGGYRNDGPRGEGGYRN 370
                 **::**********: *    * * * .*** *** . ***.*** *:.* * ***  *

VIG             REGGNRGPR-DGQQ-HNNEGGGSSAQNQRPPIDRRGPGNNQNNNQNSGPGPNKRFERQQN 475
Vig2            DGPRGEGPRNEGPRGEGYRNDGPRGEGFRGPRFNNGPSNGYENRQDNNRFGEKR--RSAQ 428
                    ..*** :* : .. ...*. .:. * *  ..**.*. :*.*:..   :**  *. :

VIG             TAPKVNDERQFPTLA 490
Vig2            KPLKVDDEAQFPTLC 443
                .. **:** *****.
```

Supplementary figure 1. CLUSTAL 2.0.8 VIG and Vig2 protein sequence alignment. Colors were used as follows: small and hydrophobic residues (AVFPMILW) are red; basic (RK) are in magenta; hydroxyl, amine (STYHCNG) and Q are green; others are grey. An alignment displays the following symbols denoting the degree of conservation observed in each column:
"*" means that the residues or nucleotides in that column are identical; ":" means that conserved substitutions have been observed; "." means that semi-conserved substitutions are observed.
