# Supplementary material for: Drosophila RISC Component VIG and Its Homolog Vig2 Impact Heterochromatin Formation
Source: PLoS One. 2009 Jul 8;4(7):e6182. doi: 10.1371/journal.pone.0006182 (PMC2703606; doi:10.1371/journal.pone.0006182)
Supplement: Figure S2 — (0.13 MB PDF) [file pone.0006182.s002.pdf]

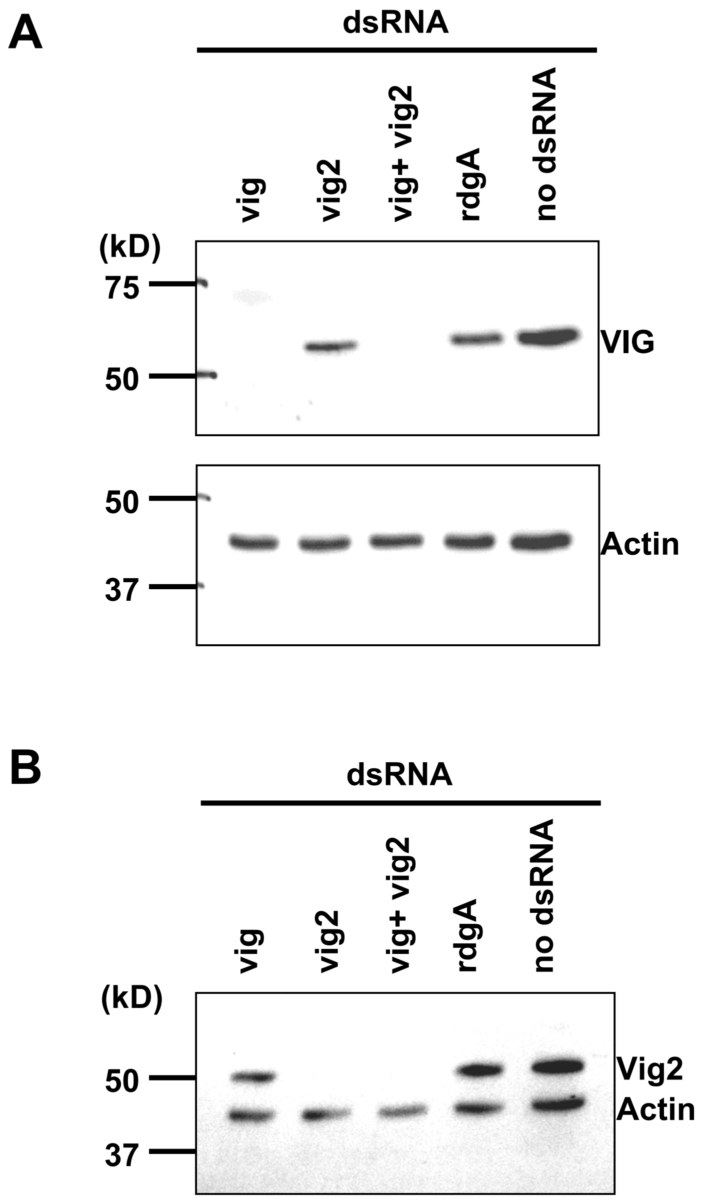

Supplementary figure 2. VIG and Vig2 antibody validation in *Drosophila* Kc cells. (A) Anti-VIG (CSH 1801) produces a band of ca 60 kD on a Western. The band is not present in cells where *vig* or *vig* and *vig2* were knocked down by RNAi. Cells were soaked with *vig* dsRNA or a mixture of *vig* and *vig2* dsRNAs as described in Clemens et al., 2000. *Vig* dsRNA was generated using primers ATTTAGGTGACACTATAGAGTGAGGACACCACACAATGG and ATTTAGGTGACACTATAGACTTGCTGCTTTCATTCACCA and RiboMAX Large Scale RNA Production System- SP6 (Promega); primers for *vig2* were ATTTAGGTGACACTATAGACGAAGAGGATGAGTCCAAGC and ATTTAGGTGACACTATAGATGTTTTCATAGCCGTTGCTG. *RdgA* gene dsRNA treatment of the cells served as a negative control. Anti-Actin was used as a loading control. (B) Anti-Vig2 (CSH 2542) produces a band ca 50 kD which disappears when *vig2* gene is knocked down.
