# Supplementary material for: Drosophila RISC Component VIG and Its Homolog Vig2 Impact Heterochromatin Formation
Source: PLoS One. 2009 Jul 8;4(7):e6182. doi: 10.1371/journal.pone.0006182 (PMC2703606; doi:10.1371/journal.pone.0006182)
Supplement: Figure S3 — (0.05 MB PDF) [file pone.0006182.s003.pdf]

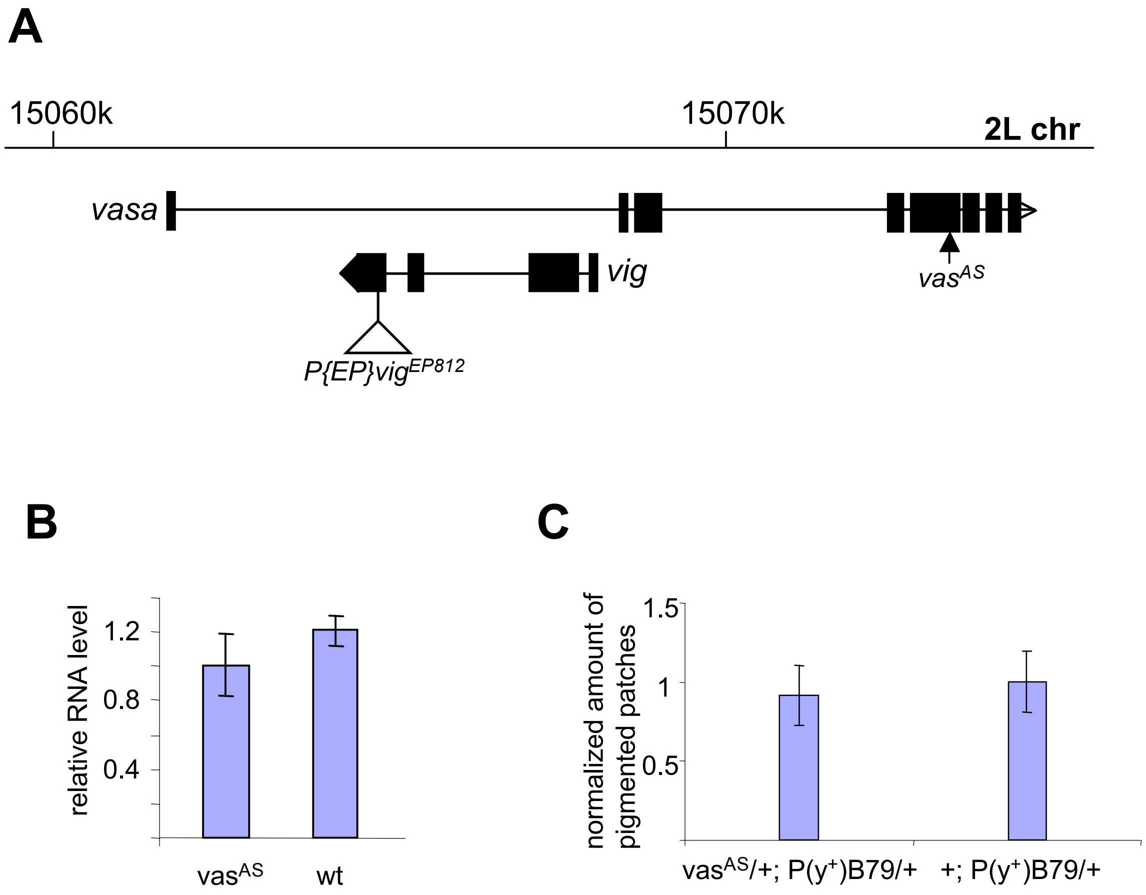

Supplementary figure 3. (A) A schematic presentation of the *vasa* gene region. Black blocks represent exons. Transcription direction is shown by horizontal arrows. The insertion site for *P{EP}vig*^{EP812} is designated by a triangle; the *vas*^{AS} mutation site is marked by a vertical arrow. (B) Levels of *vig* transcription in *vas*^{AS} and *WT* flies. Real time RT PCR was performed as described in Materials and Methods. (C) Assessment of vas^{AS} mutant as a potential PEV modifier using the variegating *yellow* reporter *P(y)B79*. Genetic crosses were performed as described in Materials and Methods. The bar graphs represent the number of pigmented patches normalized to the control as described in Fig. 2. *Vas*^{AS} does not change the variegation level of the *yellow* transgene.
