# Supplementary material for: Drosophila RISC Component VIG and Its Homolog Vig2 Impact Heterochromatin Formation
Source: PLoS One. 2009 Jul 8;4(7):e6182. doi: 10.1371/journal.pone.0006182 (PMC2703606; doi:10.1371/journal.pone.0006182)
Supplement: Figure S4 — (0.38 MB PDF) [file pone.0006182.s004.pdf]

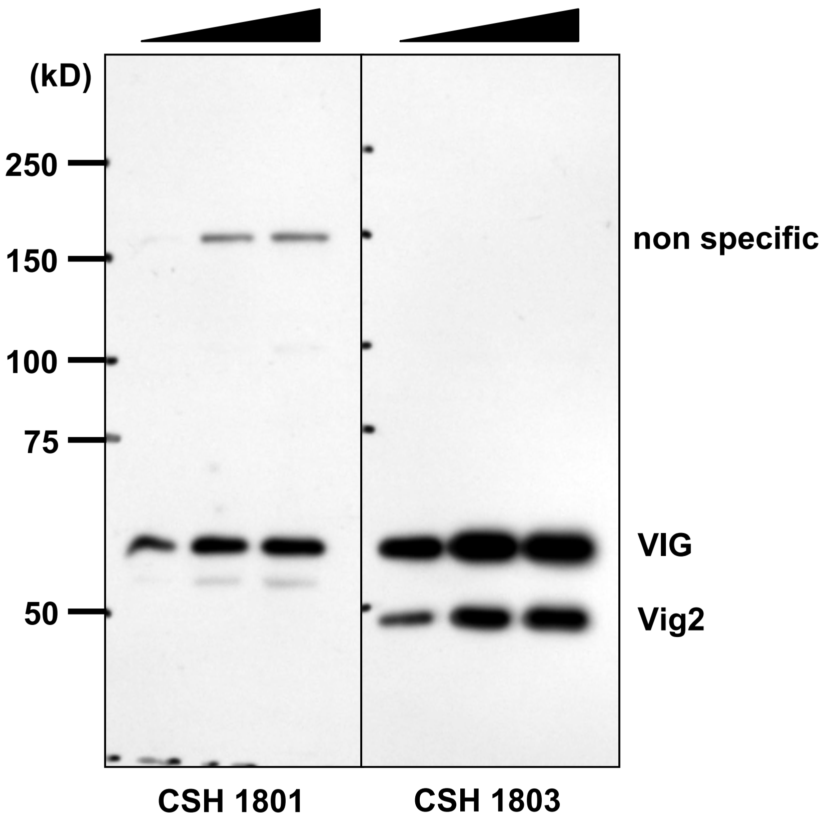

Supplementary figure 4. Comparison of two antibodies, CSH1801 and CSH1803, using protein extracts from OR salivary glands. Triangles on the top indicate increased amounts of loaded protein samples.
