# Supplementary material for: Drosophila RISC Component VIG and Its Homolog Vig2 Impact Heterochromatin Formation
Source: PLoS One. 2009 Jul 8;4(7):e6182. doi: 10.1371/journal.pone.0006182 (PMC2703606; doi:10.1371/journal.pone.0006182)
Supplement: Figure S5 — (0.06 MB PDF) [file pone.0006182.s005.pdf]

A

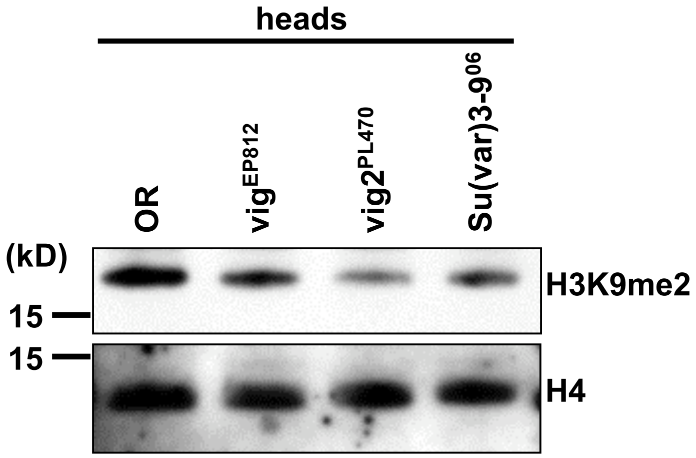

B

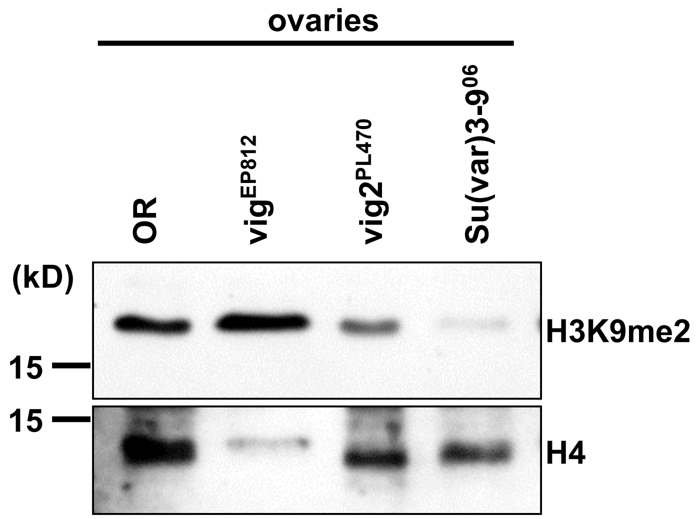

Supplementary figure 5. Assessment of H3K9 dimethylation levels in vig^{EP812} and vig2^{PL470} mutant strains by Western. (A) Total protein extracts were obtained from adult flies heads. (B) Total protein extracts were obtained from ovaries.
