# Supplementary material for: Drosophila RISC Component VIG and Its Homolog Vig2 Impact Heterochromatin Formation
Source: PLoS One. 2009 Jul 8;4(7):e6182. doi: 10.1371/journal.pone.0006182 (PMC2703606; doi:10.1371/journal.pone.0006182)
Supplement: Figure S6 — (0.04 MB PDF) [file pone.0006182.s006.pdf]

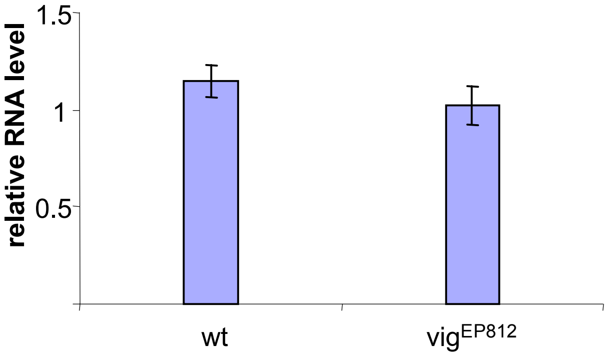

Supplementary figure 6. Results of the real time RT PCR assessing the level of *Su(var)205* (*HP1*) gene expression in a *vig* mutant. There were no significant changes observed between the *wt* and *vig*^{EP812} strain.
